# Experimentally testing the function of anal fins in the bluefin killifish, *Lucania goodei*

**DOI:** 10.1111/jfb.70293

**Published:** 2025-11-27

**Authors:** Edie Smelko, Rebecca C. Fuller

**Affiliations:** ^1^ Department of Evolution, Ecology, and Behavior, School of Integrative Biology University of Illinois Urbana Illinois USA

**Keywords:** anal fin, clasping, fitness components, Fundulidae, unpaired fins

## Abstract

Anal fins are thought to affect many functions, including swimming, sperm flow and signalling. However, there are few experimental demonstrations of these functions. We manipulated male anal fins by placing a cut in the fin. We found that cut males had lower fertilization rates than uncut males. Females were less likely to be courted and guarded by cut than uncut males. Uncut males were more likely to dominate in male/male competition. Our study suggests that anal fins have multiple important functions for fertilization and reproduction.

Nearly all fish possess anal fins, which are thought to be important for multiple functions and behaviours (Helfman et al., 2009). Anal fins are important for orientation, balance and manoeuvring in the water column (Chadwell & Ashley‐Ross, 2012; Standen & Lauder, 2005, 2007). The anal fins of many males are conspicuously coloured, suggesting that these fins are important for signalling (Borghezan et al., 2023; Breder & Rosen, 1966; Egger et al., 2011; Fuller, 2002; Theis et al., 2012; Zhou et al., 2014). Males and females of many species also differ in the size and shape of their anal fins, with males having larger anal fins than females (Davis et al., 2025; Downer‐Bartholomew & Rodd, 2025; Englmaier et al., 2022; Sumarto et al., 2020). These latter observations have led to the hypothesis that the size and shape of the anal fin affect the ability of males to fertilize eggs and control the flow of sperm in external fertilizers in addition to their possible role in signalling.

Surprisingly, there have been few experimental (i.e., manipulative) studies investigating the role of anal‐fin size, shape and structural integrity on reproduction. The notable exception is in medaka, *Oryzias latipes* (Temminck & Schlegel 1846). Fujimoto et al. (2014) took advantage of population differences in anal‐fin (and dorsal‐fin) lengths to examine mate choice for males with long anal (and dorsal) fins. They created an F2 cross between a long‐finned and a short‐finned population and then examined female mating preferences (measured as number of rejections) for males with various fin sizes. They found that males with longer anal (and dorsal) fins were less likely to be rejected by females. In another experiment, Koseki et al. (2000) compared the fertilization rates of males with full anal fins, males with the edge of the anal fins removed, males with a 50% reduction in the anal fin and males with complete removal of the anal fin. Males whose anal fins had been completely removed failed to mate. All other males mated, but males with a 50% reduction in their anal fins had reduced fertilization success compared to males with intact anal fins or males who had the edge of their anal fins trimmed. These studies provide good support for the idea that anal fins affect both male attractiveness to females and fertilization success in medaka, but the degree to which these findings are broadly generalizable is unclear.

In this study, we sought to determine whether anal fins play a role in fertilization, courtship of females, signalling in male/male competition or some combination thereof in the bluefin killifish, *Lucania goodei* (Jordan 1880). Bluefin killifish are an excellent system for studying the functional properties of anal fins. Males possess bright colouration in their anal fins, which differs from the drab colouration of females (Foster, 1967; Fuller, 2002; Mitchem et al., 2018). Males ‘flick’ these fins at females during courtship and flare them at rival males during competition (Fuller, 2001; Johnson & Fuller, 2015; McGhee et al., 2007). Additionally, the anal fins of males are approximately 55% larger than that of females and are particularly elongated on the posterior portion (Brockelsby et al., 2025; Davis et al., 2025).

To examine the effects of anal fin structural integrity on reproduction, we placed a small cut in the anal fin perpendicular to the body approximately 50% of the length of the fin. This manipulation did not shorten the fin or reduce the fin area. Rather, the purpose of the manipulation was to reduce the structural integrity of the anal fin by turning it from a single, contiguous sheet of tissue to a torn tissue with two ‘flopping’ pieces. We then compared cut males with uncut males in their ability to attract females, fertilize eggs and compete with other males. The results showed that anal fins are important to fertilization success, the likelihood of males courting and following females and the outcome of competition among males.

The fish used in this study were from a lab stock population from the Everglades (26‐Mile Bend, Broward County). The fish were originally collected with seines and dip‐nets in May 2023 and then returned to the University of Illinois, where they were housed in stock tanks in a greenhouse and fed brine shrimp and/or Daphnia daily. The greenhouse had natural light. The fish used for the current study are likely to have been second‐generation fish.

We created triads of fish with a cut male, an uncut male and a female. We size‐matched the fish within 0.5 mm, with SL ranging from 29 to 36 mm. Both males were lightly anaesthetised with a dilute solution of buffered MS‐222. For the cut male, we used scissors to place a cut from the edge of the fin to approximately halfway up the centre of the fin, perpendicular to the base (Figure 1). For the uncut male, we ran the scissors over the fin but did not place a cut. The intention of this manipulation was to ensure that the only difference between cut and uncut males was the presence or absence of an incision, whereas both groups equally experienced the potential effects of anaesthesia and scissors on the tissue. The males were then placed in 38‐L tanks where they could recover.

**FIGURE 1:**
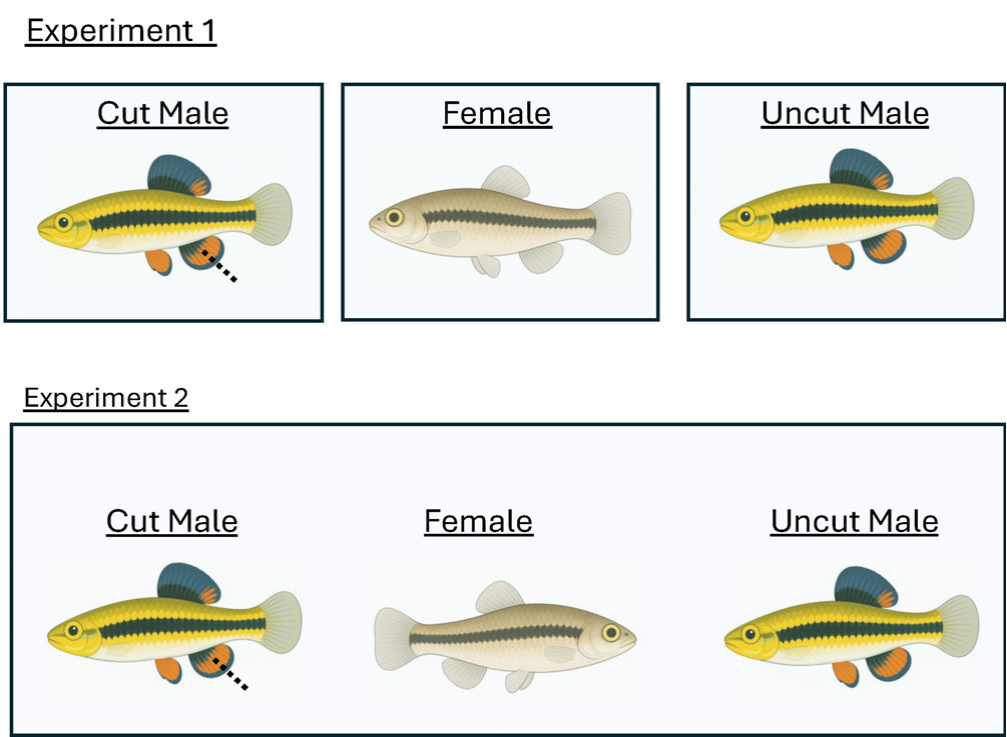
(Top) Schematic for experiment 1. The female was placed in one of the two male tanks every other day. The number of eggs laid and the proportion of eggs fertilized were recorded. The locations of the males with respect to the female were randomized. (Bottom) The three fish were placed in a large aquarium together, and interactions were observed and recorded.

We conducted two behavioural assays. In the first, we set up three 38‐L tanks adjacent to one another (Figure 1). The female was placed in the centre tank, and the two males (cut and uncut) were placed on the ends. The tank walls were kept clean of algae so that the fish could see one another. Each tank was stocked with four spawning mops, two of which were weighted so that they remained on the bottom of the tank and two of which were attached to floats so that they remained near the water surface. The fish spawn eggs on the mops and also use them for shelter. Each mop is a bundle of green yarn that roughly mimics plants. We initially set up 15 triads of fish, but two triads suffered a mortality, and the sample size was reduced to 13.

In the first assay, females were paired individually with each male on alternating days for 2 weeks. Each day, the female was transferred from her tank and placed in the tank with either the ‘cut’ or the ‘uncut’ male for 4 h, during which time the fish could interact and spawn. Afterwards, we returned the female to her home tank and then removed the spawning mops from the male's tank. We carefully sorted through the mops, removed the eggs and placed them in a small plastic tub with dilute methylene blue. We counted the eggs and then monitored them for viability over the following 4 days. Therefore, from this experiment, we could measure female mating preference as the number of eggs spawned with the cut versus the uncut male and assess the fertilization success of cut and uncut males.

In the second assay, we placed all three fish into a long 76‐L tank where they were free to interact. We watched the fish every other day for 15‐min time periods until we had completed three observation periods. During each observation, we recorded the number of attacks towards the competing male and towards the female, as well as the number of chases towards the competing male and the number of times the male followed the female. We also recorded the number of fin flares, but we were unable to distinguish whether these were directed towards the competing male or the female. Past work indicates that the vast majority of fin flares are directed towards the competing male (Fuller, 2001). We also recorded the number of times each male courted the female. All observations took place over a 15‐min period in the early morning, when the fish were the most active. Tanks were covered with a black curtain, and observations were made from a distance so as not to disturb the natural behaviour of the fish. Two triads suffered a mortality between the first and second assays. Therefore, our sample size was reduced to 11 triads for the second assay.

We used linear models to examine the effects of the anal fin manipulation on female preference (number of eggs spawned), fertilization success (% eggs alive after 1 week) and male dominance. To do this, we used a simple mixed model where we examined the effects of anal fin treatment (cut versus intact) on the dependent variable. We include the triad ID as a random effect. We used linear models with a normal distribution to test the effects of the anal fin treatment on female preference (number of eggs with each male) and male dominance (number of chases, attacks, female following bouts, courtship bouts and spawning events). We tested these models using 'anova' from the lmerTest package with the default Satterthwaite degrees of freedom in the denominator. For fertilization success, we used a generalized linear model with a binomial distribution treating triad ID as a random effect. We tested this model using ‘Anova’ in the car package in R. The raw data and R codes can be found at [https://doi.org/10.5061/dryad.tmpg4f5br].

The anal‐fin treatment (cut versus uncut anal fins) affected multiple aspects of behaviour and mating (Figure 2). In the first experiment, fertilization success was significantly higher in uncut males than in cut males (90% versus 80%, $X_{1}^{2}=8.81$, *p* = 0.0030). However, there was no statistically significant difference in the number of eggs laid with cut versus uncut males (*F*_1,12_ = 3.62, *p* = 0.0813).

**FIGURE 2:**
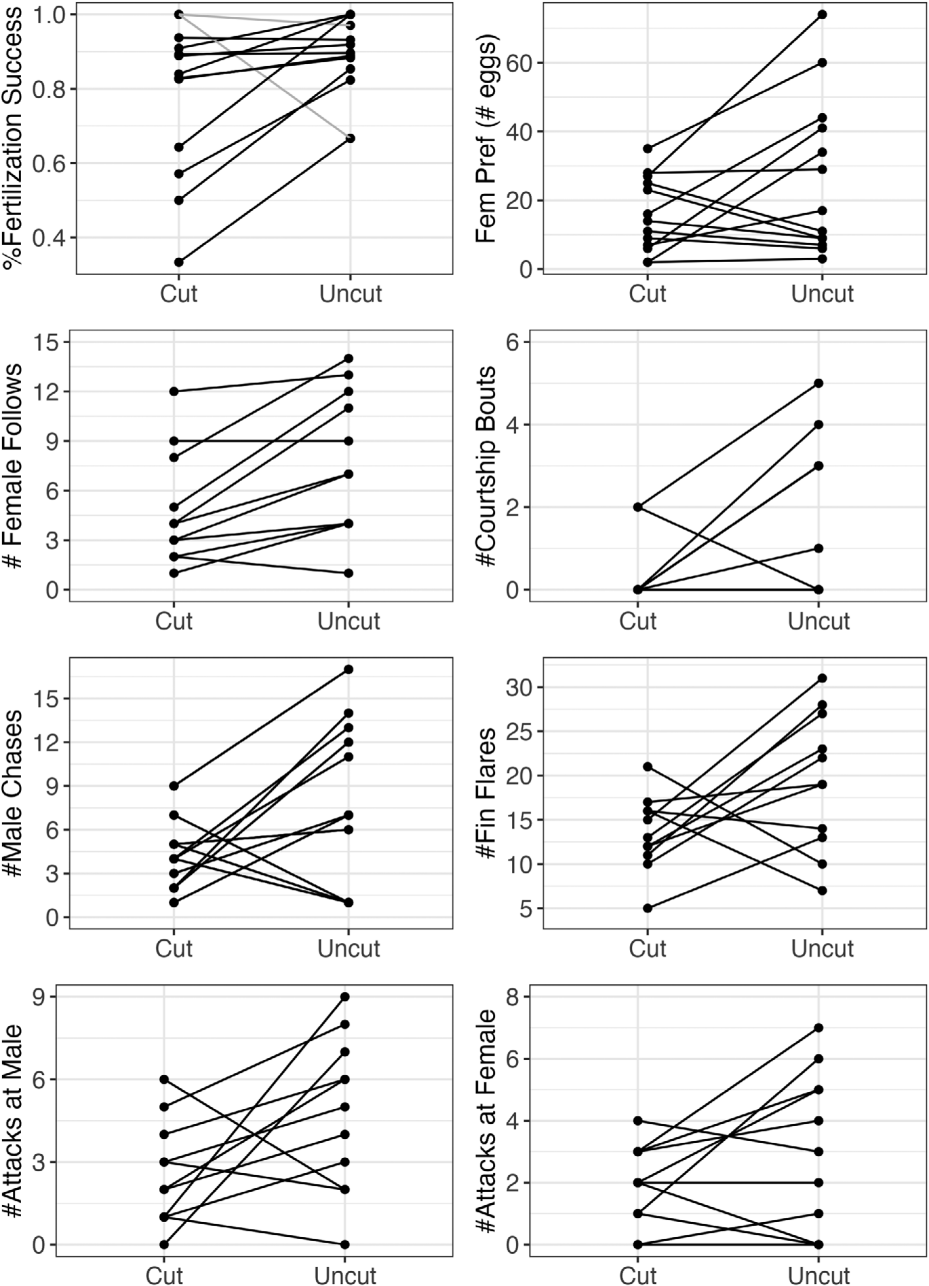
Response variables as a function of cut versus uncut anal fins. Lines combine paired males for a given triad of fish. For fertilization success, the two lines with grey colour had clutch sizes of two eggs for the cut males.

In the second experiment, where the three fish could freely interact, the uncut males were more likely to follow and court the female (follows: *F*_1,10_ = 13.03, *p* = 0.0048; courtship bouts: *F*_1,10_ = 5.60, *p* = 0.0396). Uncut males also dominated in competition. The uncut male chased, attacked and flared his fins more than the cut male (chases: *F*_1,20_ = 4.72, *p* = 0.0421; attacks: *F*_1,10_ = 4.64, *p* = 0.0436; fin flares: *F*_1,20_ = 4.94, *p* = 0.03791). However, there were no statistically significant effects of the manipulation on the propensity of males to attack the female (*F*_1,10_ = 2.68, *p =* 0.1329). Differences in the denominator degrees of freedom in the *F*‐tests were due to differences in variance among triads and the effects via Satterthwaite's degrees of freedom (Satterthwaite, 1946). The random effect of triad was dropped from the models when triads accounted for no variance.

This study provides strong support for the idea that anal fins are multifunctional traits that affect many fitness components in bluefin killifish, *Lucania goodei*. We found evidence that altering the structural integrity of the anal fin altered the ability of the males to successfully fertilize eggs in pair spawnings and the likelihood that they would court and follow females in free‐spawning competitive trials. Uncut males also fared better in competition over cut males. To the best of our knowledge, this is just the second fish system using an experimental manipulation to investigate the effects of structural anal‐fin properties on fitness components. Our findings are very similar to those found in medaka, *Oryzias latipes* (Fujimoto et al., 2014; Koseki et al., 2000). In medaka, alterations to the anal fin affected male fertilization success, and males with larger anal fins were less likely to be rejected by females.

This study lacks the ability/power to clearly discern the roles of female choice versus male competition on the fitness consequences of intact anal fins. The first experiment was a one‐way choice study where females could choose whether or not to spawn, which is a conservative choice assay (Houde, 1997). This experimental procedure has been effective in past work on the evolution of conspecific preferences (Gregorio et al., 2012; Kozak et al., 2015; St John & Fuller, 2019, 2021). In the free‐swimming assay, uncut males fared better than cut males in their ability to follow and court the female. Past work has indicated that the ability to court and follow the female is due to a combination of male competitive ability and female preferences (McGhee et al., 2007). Males with uncut fins also outcompeted males with cut fins. Collectively, these results suggest that functional anal fins are important to the ability of males to fertilize eggs and obtain spawnings.

The degree to which these results apply broadly to other fishes is unknown. Medaka and bluefin killifish occur in the same superorder (Atherinomorpha) but are members of separate orders, with bluefin killifish in Cyprinodontiformes and medaka in Beloniformes (Nelson et al., 2016). In addition, both of these species are pair spawners where males use their anal and dorsal fins to clasp the female (Breder & Rosen, 1966; Foster, 1967). The order in which these anal fin functions evolved is unknown and is beyond the scope of this study. However, we speculate that anal fins first evolved for stabilization (i.e., to prevent rolling and allow for quick turns) and then subsequently became used to control the direction and flow of sperm, with signalling functions evolving later. Nearly all fish have anal fins. Juveniles rapidly develop anal fins early in development, suggesting their importance to non‐reproductive behaviours. Although sexual dimorphism in anal‐fin traits is common in teleosts, it is not universal. The comparative pattern between sexual dimorphism and anal‐fin functions is unknown.

Finally, we note that our experiment examined the effects of anal fin structural integrity, but it did not examine the effect of anal fin size per se. We were reticent to reduce the size of the anal fin by trimming it, as this would remove the black border, which is important for signalling among males (Johnson & Fuller, 2015). Going forward, investigations into the size and shape of anal fins might benefit from utilizing genetic variation among populations (if present) and/or environmentally induced variation. Work in other fundulids has shown that high temperatures can cause an increase in anal fin ray number (Fahy, 1979), which may alter adult anal fin size. Fujimoto et al. (2021) have also found seasonal variation in the level of sexual dimorphism in anal fin size in medaka. If this variation is present in fundulids, then it may allow for investigations into the costs and benefits of anal fin size and shape. Other approaches might involve identifying specific loci involved in fin dimorphism (e.g., Kawajiri et al., 2014) and subsequent direct genomic alterations via *crispr*. Indeed, crispr has already been used in medaka and zebrafish to alter anal fin properties (Adachi et al., 2024; Ansai et al., 2021). Such approaches may become central to future efforts to investigate the evolution of sexual dimorphism in anal fins.

## AUTHOR CONTRIBUTIONS

R.C. Fuller designed the study, collected fish, and analyzed the data. E. Smelko conducted the assays. Both authors wrote the manuscript.

## FUNDING INFORMATION

The study was funded by the University of Illinois at Urbana–Champaign.

## CONFLICT OF INTEREST STATEMENT

The authors declare no conflicts of interest.

## Data Availability

The data and R codes can be found at Dryad. https://doi.org/10.5061/dryad.tmpg4f5br.
